# Editorial: Rehabilitation and regeneration in orthopaedic: from cellular regulation to innovative medical technologies

**DOI:** 10.3389/fcell.2025.1633691

**Published:** 2025-06-05

**Authors:** Weidan Wang, Ye Li, Jiannan Li, Weihao Yuan, Dewei Zhao

**Affiliations:** ^1^ Affiliated Zhongshan Hospital of Dalian University, Dalian, China; ^2^ Department of Rehabilitation Sciences, The Hong Kong Polytechnic University, Kowloon, Hong Kong SAR, China; ^3^ University of Texas, Dallas, TX, United States; ^4^ School of Dentistry, University of California, Los Angeles, Los Angeles, CA, United States

**Keywords:** orthopeadic, rehabilitation, regeneration, cellular regulation, biomaterials

## Introduction

Orthopaedic rehabilitation and regeneration represent a transformative frontier in musculoskeletal medicine, where cutting-edge biological insights converge with advanced engineering innovations to reshape clinical development. This Research Topic compiles articles investigating the latest developments in cellular regulation, non-coding RNA biology, stem cell co-culture systems, and innovative medical technologies for orthopaedic applications. Additionally, these studies enhance the understanding of orthopaedic regeneration and provide practical strategies for clinical implementation.

## Bridging research insights and clinical imperatives

(1) Non-coding RNA-mediated osteogenic regulation (Aranguren et al.). This study illustrates how circular RNAs, snoRNAs, and piRNAs coordinate osteogenic differentiation *via* epigenetic modulation of RUNX2 and BMP/SMAD pathways. The therapeutic potential of these non-coding RNAs for bone fracture healing and tissue regeneration is also emphasized, offering novel insights into the translational applications of RNA biology in orthopaedics.
(2) 3D bioprinting and stem cell engineering (Liu et al.). A co-culture platform combining STRO1+ human gingival mesenchymal stem cells (GMSCs) with human umbilical vein endothelial cells (HUVECs) is presented to create vascularized osteogenic constructs. This research improves the understanding of how 3D co-culture systems can be utilized to develop more effective cell therapy strategies for bone tissue engineering.
(3) Immunomodulatory MSC therapies (Zhang et al.). The authors summarize the paracrine signals of bone marrow MSCs, which play a crucial role in immune regulation and inflammation suppression. They discuss how MSC-derived exosomes and cytokines can modulate immune responses and promote cartilage repair, providing a theoretical foundation for the clinical application of MSC transplantation in osteoarthritis (OA) treatment.
(4) Laser-engineered bioceramics (Daskalova et al.). Femtosecond laser texturing is applied to create hierarchical microchannels on freeze-foamed TCP/ZrO_2_ hybrids. This novel research demonstrates how ultra-short laser processing can enhance the surface properties and cellular affinity of 3D bioceramic constructs, highlighting the potential of this technology for developing next-generation bone substitutes.

## Clinical translation: from bench to bedside

This Research Topic underscores the necessity of hybrid solutions combining biological precision with engineered reliability, which aligns with the work of vascularized bone regeneration strategies, biofunctionalized bone grafts and additive manufacturing techniques by Zhao et al. (2016), Zhao et al. (2025). Furthermore, these articles contribute to a deeper understanding of the cellular and molecular mechanisms governing bone healing and regeneration, showing potential translation of these innovative technologies in orthopaedic clinical practice. From the regulatory roles of non-coding RNAs to the development of advanced biomaterials and cell therapy strategies, the research presented in this Research Topic highlights the interdisciplinary nature of current orthopaedic science. As the complexities of biology mechanisms and materials science are continually revealed, we are edging closer to realize more effective and personalized approaches to rehabilitation and regeneration in orthopaedic application.
